# Comparing Open and Robotic Unilateral Transversus Abdominis Release in Incisional Hernias With a Lateral Component: A Single Center Retrospective Study

**DOI:** 10.3389/jaws.2024.13256

**Published:** 2025-02-03

**Authors:** Stijn Van Hoef, Hasan H. Eker, Frederik Berrevoet, Mathias Allaeys

**Affiliations:** ^1^ Department of Abdominal Surgery, Virga Jessa–Sint–Trudo, Hasselt-Sint-Truiden, Belgium; ^2^ Department of General and Hepatobiliary (HPB) Surgery and Liver Transplantation, Ghent University Hospital, Ghent, Belgium

**Keywords:** hernia, robotic abdominal wall repair, TAR, comparative analysis, lateral abdominal wall

## Abstract

**Introduction:**

Lateral hernias are often more challenging to correct when compared to midline defects, due to the anatomic boundaries of the bony pelvis, retroperitoneum, and costal margin. With the insurgence of robot assisted abdominal wall surgery, these defects have been found more manageable through a minimal invasive repair. In this study, we aim to present our short-term results of incisional hernia repair with a lateral component requiring a unilateral transversus abdominis release, through open surgery versus robot assisted.

**Methods:**

A retrospective analysis was performed of our robotic and open abdominal wall repairs of lateral hernias, where a unilateral transversus abdominis release was performed, between January 2017 and December 2023. Patient, hernia and perioperative details are reported.

**Results:**

54 patients in the open group versus 10 patients in the robotic group were included. Hernia width and hernia surface area were higher in the open group, but not significant. Operation time was similar between open and robotic procedures. In-hospital complications, surgical site infection and clinical seroma rate during the first 30 postoperative days were similar in both groups. There was a clear difference in length of stay, in favor of the robotic group.

**Discussion:**

In our limited series, a robotic approach seems safe and feasible when faced with large lateral hernias. Short-term results show a shorter length of stay using the robotic approach, with no significant difference in short term complications, specifically SSI-rate. However, conclusions are limited due to the low number of patients and additional studies should be performed to account for long term recurrence and increase included patient number.

## Introduction

Lateral hernias, as defined by the European Hernia Society [1], are often more challenging to correct when compared to midline defects. Repair is technically demanding due to anatomic boundaries of the bony pelvis, retroperitoneum, and costal margin. They are predominantly secondary to prior subcostal or flank incisions, ostomy sites, traumatic abdominal wall injury, or trocar sites. True congenital lateral wall defects are exceedingly rare, with very few reported cases [2, 3]. These defects can be repaired through both an open or minimally invasive fashion, with mesh placement either intra-peritoneal, pre-peritoneal or onlay, bearing in mind the principles of tension free fascial approximation and adequate mesh overlap. To achieve this, frequently component separation is required, especially if combined with a midline defect. Originally devised by Novitsky in 2012 [4], the transversus abdominis release (TAR) builds seamlessly on the known Rives-Stoppa retromuscular plane, and provides not only medial advancement of the anterior fascia in the midline, but also opens the pre-peritoneal plane for a wide mesh overlap.

The introduction of minimally invasive tools in abdominal wall reconstruction has led to, as in other surgical specialties, reduced length of stay, earlier return to work and fewer wound complications [5]. However, due to its technical complexity and poor ergonomics, laparoscopic repair never found its way into complex abdominal wall repair [6]. With the increased availability of robotics, this has changed, and more and more difficult abdominal wall defects, with large fascial defects and hard to reach areas, are repaired by a robot assisted technique. Other series have already shown that the robotic approach is safe and feasible, showing good short term outcomes [6–10]. However, these are mainly based on midline hernias. To see whether these findings are also applicable for lateral hernias, we retrospectively reviewed our own series.

## Methods

We retrospectively reviewed our robotic and open abdominal wall repairs of lateral hernias, with or without medial component where a unilateral TAR was performed. All the procedures were performed between January 2017 and December 2023, by 3 dedicated hernia surgeons. Informed consent was obtained from all patients. The study was approved by UZ Gent Ethics committee under number BN-09636. Medical records were examined for patient characteristics, hernia characteristics, perioperative and postoperative details, and short-term post-operative outcome. Patient data included age, body mass index (BMI), gender, diabetes, smoking status, presence of pulmonary disease, use of immunosuppressants or corticosteroids, history of aortic aneurysm of collagen related disease and American Society of Anesthesiologist (ASA) class. The included hernia characteristics were prior hernia surgery, hernia defect size (width and surface area), mesh size, type of hernia repair, and hernia location based on the European Hernia Society (EHS) classification. Perioperative details included wound class, mesh type, mesh position, mesh size (width – length – surface area) and intraoperative surgical complications. Post-operative details included length of stay (LOS), in hospital complications and 30-day morbidity (seroma, SSI, recurrence).

The statistical analysis included a univariate analysis performed on categorical variables to elucidate risk factors for hernia recurrence. Chi-squared test was used to determine the significance of each risk factor and to delineate complications by type of repair. Continuous variables were compared using an independent-samples t-test or ANOVA test in cases of non-normal distribution. Significance was defined as *p* < 0.05. Standard deviation was calculated for hernia defect, mesh size, operation time and LOS. Pre-, peri- and post-operative data was prospectively maintained in a RedCap database. Data analysis was carried out with SPSS^®^ Statistics (IBM, Armonk, New York, United States).

The robotic-assisted surgical procedures were performed with the DaVinci Xi or X system (Intuitive Surgical, Sunnyvale, California, United States). The surgical procedure is similar as previously described by others [8].

## Results

A total of 54 patients in the open TAR group and 10 patients in the robotic TAR group were included. Patient demographics are summarized in Table 1. No significant differences between patient groups were noted regarding age, sex, BMI, smoking status, diabetes, chronic use of corticosteroids, history of aortic aneurysm and collagen related disease. There were significantly more patients with a higher ASA-score in the open TAR-group as well as patient on immunosuppressants.

**TABLE 1:** Patient demographics.

	Open TAR	Robot TAR	*p*-value
N	54	10	
Age, years (mean ± SD)	61 ± 12	62 ± 7	0.782
Gender			
Male	31 (57.4%)	5 (50.0%)	0.664
Female	23 (42.6%)	5 (50.0%)	
BMI (mean ± SD)	28.51 ± (4.62)	29.55 ± (5.42)	0.529
ASA-score			
1	0	0	0.056
2	21 (38.9%)	8 (80.0%)	
3	32 (59.2%)	2 (20.0%)	
4	1 (1.9%)	0	
Smoking			
Never smoked	19 (35.1%)	5 (50.0%)	0.304
Ex-smoker (>12 months stopped)	28 (51.9%)	4 (40.0%)	
Occasional smoker	1 (1.9%)	1 (10.0%)	
Daily smoker	6 (11.1%)	0	
Pulmonary disease	9 (16.7%)	1 (10.0%)	0.594
Diabetes			
Type I	1 (1.9%)	0	0.664
Type II	12 (22.2%)	1 (10.0%)	0.378
Immunosuppressants	25 (46.3%)	1 (10.0%)	**0.032**
Corticosteroids (chronic)	2 (3.7%)	0	0.536
History of aortic aneurysm	1 (1.9%)	0	0.664
Collagen related disease	1 (1.9%)	0	0.664

Values marked in bold are statistically significant.

Hernia characteristics are shown in Table 2. No differences between groups were seen regarding hernia width, hernia surface area, prior hernia surgery, EHS classifications, CDC wound class, mesh type or position. Mesh size was significantly larger in the open TAR group. There were no intra-operative complications in the robotic group, there were two bowel lesions and one ureteral injury in the open TAR group.

**TABLE 2:** Hernia characteristics.

	Open TAR	Robot TAR	*p*-value
N	54	10	
Hernia Width	11.82 ± 6.11	9.83 ± 7.65	0.366
Hernia Surface Area	166.26 ± 141.74	104.79 ± 110.51	0.199
Recurrent Hernia	16 (29.6%)	2 (20.0%)	0.534
EHS Classification^a^			
M1	7 (13.0%)	0	0.228
M2	22 (40.7%)	3 (30.0%)	0.523
M3	18 (33.3%)	3 (30.0%)	0.837
M4	8 (14.8%)	1 (10.0%)	0.687
M5	5 (9.3%)	0	0.316
No midline component	23 (42.6%)	5 (50.0%)	0.546
L1	10 (18.5%)	1 (10.0%)	0.512
L2	33 (61.1%)	7 (70.0%)	0.595
L3	15 (27.8%)	3 (30.0%)	0.886
L4	14 (25.9%)	1 (10.0%)	0.275
CDC^b^			
I	46 (85.2%)	10 (100.0%)	0.429
II	4 (7.4%)	0	
III	4 (7.4%)	0	
Mesh Size			
Width	32.22 ± 8.76	21.90 ± 8.35	**<0.001**
Length	38.52 ± 10.72	23.90 ± 12.07	**<0.001**
Surface Area (cm^2^)	1302.19 ± 679.38	597.30 ± 462.27	**0.003**
Intraoperative Complications			
Bowel Lesion	2 (3.7%)	0	0.156
Ureteral Injury	1 (1.9%)	0	0.210

^a^ As stated by Muysoms et al. [1].

^b^ According to the Center for Disease Control and Prevention (CDC) classification.

Values marked in bold are statistically significant.

Outcome data are displayed in Table 3. Operation time was similar between open and robotic procedures. In-hospital complications, surgical site infections (SSIs) and clinical seroma rate during the first 30 postoperative days were similar in both groups. There was a clear difference in LOS, where the robotic group had a significant shorter LOS compared to the open group (Open group: 6.94 ± 5.07 vs. Robotic group: 1.50 ± 0.97, *p* = 0.001). Only one major postoperative complication occurred (Clavien–Dindo grade III and above), in the open group. In the 30-day follow-up period there were no mesh removals, nor early recurrences.

**TABLE 3:** Outcome characteristics.

	Open TAR	Robot TAR	*p*-value
N	54	10	
Operation Time (in minutes)	185.15 ± 85.04	160.40 ± 69.26	0.389
Hospital stay	6.94 ± 5.07	1.50 ± 0.97	**0.001**
In hospital complications			
None	41 (75.9%)	10 (100.0%)	0.082
Hemorrage	0	0	
SSI	2 (3.7%)	0	0.536
Prolonged Ileus	6 (11.1%)	0	0.268
Medical Complications	10 (18.5%)	1 (10.0%)	0.512
In hospital Clavien Dindo			
<II	46 (85.1%)	10 (100.0%)	0.621
II	7 (13.0%)	0	
IIIa	0	0	
IIIb	1 (1.9%)	0	
30 Days Clinical Seroma Rate	5 (9.3%)	0	0.316
30 days SSI	5 (9.3%)	0	0.316
Mesh removal	None	None	
30 days Recurrence	None	None	

Values marked in bold are statistically significant.

## Discussion

The impact of minimally invasive approach during abdominal wall repair on LOS has already been shown with the introduction of laparoscopic hernia repair [11], and our series, together with others [6–8, 12], shows a similar effect of the robotic approach, even in more complex lateral hernia cases. We were unable to detect any differences in SSI- or complication-rate between both groups, and this despite the higher ASA-score in the open group and despite the significant difference in used mesh size. Several other groups have stated a similar trend [6, 8], where a large recent systematic review by Bracale et al. confirmed this finding [13]. However, it should be noted that in their analysis, this effect might be due to inclusion of hybrid procedures. Our SSI-rate in the open group is similar to that reported by the large systematic review by Vasavada et al. [14], and this despite the high rate of immunosuppressant-use in this group. As these procedures were all performed in a liver-transplantation center, we are faced with a high number of incisional hernias after liver transplantation. However, it seems that immunosuppressant-use did not significantly impact our SSI-rate [15].

In contrast to other similar studies [6, 8, 9, 13], our operation time did not significantly differ between the open and robotic group. This might be in part by the fact that all procedures were performed by dedicated (robotic) hernia surgeons, bypassing any learning curve. However, another explanation might be that in the open procedure, the hernia surface area was higher and placed mesh was larger, resulting in a larger and more laborious dissection during these procedures. With only a follow-up of 30 days, no statement can be made about technique superiority regarding recurrence rate.

As our series shows, a robotic approach is safe and feasible when faced with incisional hernias with a lateral component. It provides a shorter LOS, with no difference in short-term peri- and post-operative outcomes. As our SSI-rate does not differ between both group, one can argue, that this lower LOS can be attributed to less post-operative pain, and earlier mobilization, similar to what Carbonell et al proposed during robotic retromuscular ventral hernia repair [16]. However, open hernia repair often involves additional wound drainage, which might be reflected in the larger mesh used, involving larger dissection planes, and therefore this could impede early discharge. A larger prospective study with longer follow-up is needed, to confirm these findings and to be able to compare recurrence rates.

We do acknowledge that our study has several limitations. As this series is a retrospective study, it suffers from the limitations thereof. Furthermore, according to our surgical experience with open and robotic TAR, patient selection bias might be present, which we attribute to an early robotic experience. Furthermore, we suffer from a relative small sample size and unevenly distributed groups. Therefore the true effect on LOS might be overestimated as well as it makes proper cohort matching, as well as sub-analysis of different parameters impossible.

## Data Availability

The raw data supporting the conclusions of this article will be made available by the authors, without undue reservation.
